# Large extracellular vesicles derived from human regulatory macrophages (L-EV_Mreg_) attenuate CD3/CD28-induced T-cell activation in vitro

**DOI:** 10.1007/s00109-023-02374-9

**Published:** 2023-09-19

**Authors:** Martin Albrecht, Lars Hummitzsch, Rene Rusch, Christine Eimer, Melanie Rusch, Katharina Heß, Markus Steinfath, Jochen Cremer, Fred Fändrich, Rouven Berndt, Karina Zitta

**Affiliations:** 1 Department of Anesthesiology and Intensive Care Medicine, University Hospital of Schleswig-Holstein, Kiel, Germany; 2 Clinic of Cardiovascular Surgery, University Hospital of Schleswig-Holstein, Kiel, Germany; 3 Department of Pathology, University Hospital of Schleswig-Holstein, Kiel, Germany; 4 Clinic for Applied Cell Therapy, University Hospital of Schleswig-Holstein, Kiel, Germany

**Keywords:** Macrophages, Large extracellular vesicles, T-cell activation, Phosphatidylserine

## Abstract

**Abstract:**

Macrophages belong to the innate immune system, and we have recently shown that in vitro differentiated human regulatory macrophages (Mreg) release large extracellular vesicles (L-EV_Mreg_) with an average size of 7.5 μm which regulate wound healing and angiogenesis in vitro. The aim of this study was to investigate whether L-EV_Mreg_ also affect the CD3/CD28-mediated activation of T-cells. Mreg were differentiated using blood monocytes and L-EV_Mreg_ were isolated from culture supernatants by differential centrifugation. Activation of human T-cells was induced by CD3/CD28-coated beads in the absence or presence of Mreg or different concentrations of L-EV_Mreg_. Inhibition of T-cell activation was quantified by flow cytometry and antibodies directed against the T-cell marker granzyme B. Phosphatidylserine (PS) exposure on the surface of Mreg and L-EV_Mreg_ was analyzed by fluorescence microscopy. Incubation of human lymphocytes with CD3/CD28 beads resulted in an increase of cell size, cell granularity, and number of granzyme B–positive cells (*P* < 0.05) which is indicative of T-cell activation. The presence of Mreg (0.5 × 10^6^ Mreg/ml) led to a reduction of T-cell activation (number of granzyme B–positive cells; *P* < 0.001), and a similar but less pronounced effect was also observed when incubating activated T-cells with L-EV_Mreg_ (*P* < 0.05 for 3.2 × 10^6^ L-EV_Mreg_/ml). A differential analysis of the effects of Mreg and L-EV_Mreg_ on CD4^+^ and CD8^+^ T-cells showed an inhibition of CD4^+^ T-cells by Mreg (*P* < 0.01) and L-EV_Mreg_ (*P* < 0.05 for 1.6 × 10^6^ L-EV_Mreg_/ml; *P* < 0.01 for 3.2 × 10^6^ L-EV_Mreg_/ml). A moderate inhibition of CD8^+^ T-cells was observed by Mreg (*P* < 0.05) and by L-EV_Mreg_ (*P* < 0.01 for 1.6 × 10^6^ L-EV_Mreg_/ml and 3.2 × 10^6^ L-EV_Mreg_/ml). PS was restricted to confined regions of the Mreg surface, while L-EV_Mreg_ showed strong signals for PS in the exoplasmic leaflet. L-EV_Mreg_ attenuate CD3/CD28-mediated activation of CD4^+^ and CD8^+^ T-cells. L-EV_Mreg_ may have clinical relevance, particularly in the treatment of diseases associated with increased T-cell activity.

**Key messages:**

- Mreg release large extracellular vesicles (L-EV_Mreg_) with an average size of 7.5 µm
- L-EV_Mreg_ exhibit phosphatidylserine positivity
- L-EV_Mreg_ suppress CD4^+^ and CD8^+^ T-cells
- L-EV_Mreg_ hold clinical potential in T-cell-related diseases

## Introduction

Extracellular vesicles (EV) are small structures ranging from nano- to micrometer in size that are released by almost all cell types [1, 2]. EV contain lipids, proteins, and RNAs, making them an efficient way to transfer functional cargoes and signals between cells [1–4]. Growing evidence suggests that EV play a critical role in complex communication among different cell types [2, 5]. Interestingly, large extracellular vesicles (L-EV), which range in diameter from 1 to 10 μm, have recently gained attention as potential sources of bioactive molecules and mediators of cell communication and angiogenesis [6, 7].

We have shown that human monocyte-derived regulatory macrophages (Mreg) contain and release pro-angiogenic proteins and produce large amounts of L-EV_Mreg_ as part of their differentiation process [8]. These L-EV_Mreg_ display an average size of 7.5 μm and an average volume of 0.2 pl. They carry distinct vesicular surface markers that characterize them as typical EV (LAMP-1, CD9, CD63, and CD81; based on MISEV guidelines 2018 [9]). In vitro, L-EV_Mreg_ are able to promote wound healing and have a positive effect on several parameters of angiogenesis which suggests that they could bear therapeutic potential for the treatment of chronic wounds and ischemia-associated diseases such as peripheral arterial occlusive disease [8].

It has been described that macrophages, among their numerous other functions, are also able to communicate with and regulate immune cells [10, 11]. In this context, Mreg can inhibit the activation of immune cells and have been successfully employed as therapeutic approach in recipients of kidney transplant to minimize the burden of general immunosuppression [12]. However, whether L-EV_Mreg_ are also able to regulate immune cells and which mechanisms are involved is still completely unclear. Here, we show that L-EV_Mreg_ are generated during the in vitro differentiation of monocytes to Mreg. L-EV_Mreg_ attenuate the CD3/CD28-induced activation of CD4^+^ and CD8^+^ T-cells and could therefore mediate immunomodulatory functions in vivo.

L-EV_Mreg_ can be easily isolated from Mreg cultures by differential centrifugation and could be therapeutically valuable for many different clinical indications and diseases that are associated with a dysregulated immune response (i.e., increased T-cell activity).

## Materials and methods

### Mreg differentiation and isolation of L-EV_Mreg_

**Fig. 1:**
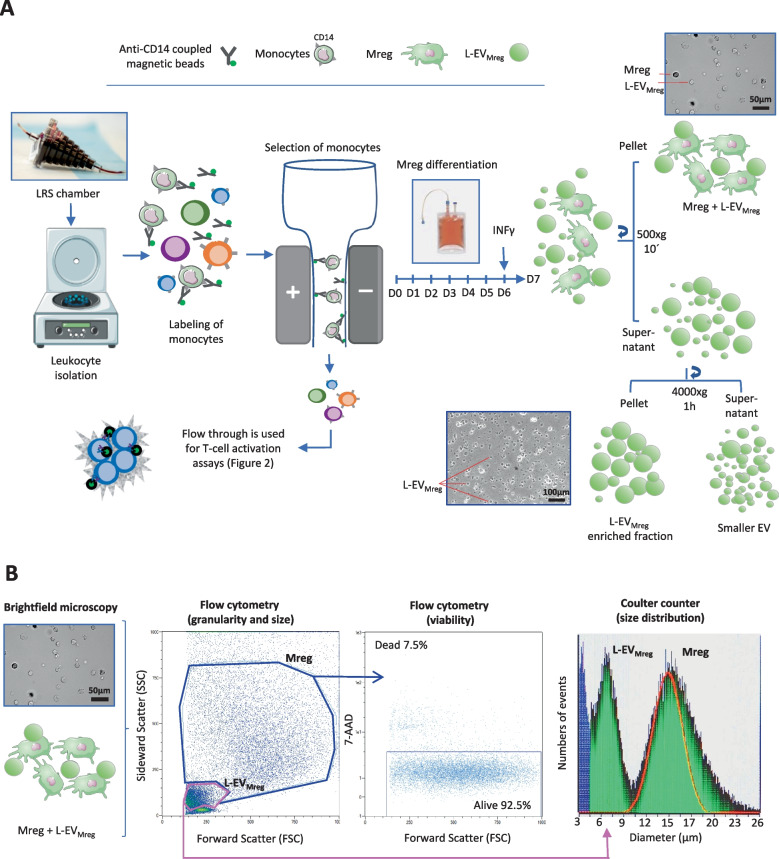
Isolation and basic characterization of Mreg and L-EV_Mreg_. **A** Schematic representation of the differentiation of Mreg from human monocytes and subsequent purification of L-EV_Mreg_. **B** Results of the basic characterization of one representative Mreg and L-EV_Mreg_ fraction by flow cytometry and Coulter counter–based analysis. Mreg and LEV_Mreg_ can be distinguished as two distinct populations based on their granularity and size

The study was approved by the local Ethics Committee of the University Medical Center Schleswig-Holstein, Kiel, Germany (protocol identification: D519/18 and D518/13). Peripheral blood mononuclear cells (PBMC) were obtained from leukocyte reduction system (LRS) chambers provided by the Department of Transfusion Medicine (University Hospital of Schleswig-Holstein, Kiel, Germany). Monocytes were isolated and differentiated to Mreg as described earlier [8] and depicted in Fig. 1A. Briefly, PBMC were purified through a Ficoll-Paque PLUS gradient (GE Healthcare, Chicago, USA) and monocytes were recovered using a CD14 magnetic bead cell sorting system (Miltenyi, Bergisch Gladbach, Germany) following the manufacturer’s protocol. The isolated CD14^+^ monocytes were cultivated in cell cultivation/differentiation bags (Miltenyi) at 0.83 × 10^6^ cells/ml with RPMI 1640 medium containing GlutaMax (GIBCO, Billings, MT, USA) supplemented with 10% human AB-serum (Access Biological, Vista, CA, USA) and 2500 IU/ml human M-CSF (R&D Systems, Wiesbaden, Germany). After 6 days in culture, 500 IU/ml of human interferon (IFN) γ (R&D Systems, McKinley Place MN, USA) was added to the cultures and cells were incubated for additional 24 h. On day 7, Mreg were harvested and separated from culture medium by centrifugation (500xg for 10 min at room temperature; Fig. 1A). The pellets containing Mreg were resuspended in PBS and subjected to further analyses. The remaining culture supernatant containing EV was further centrifugated at 4000xg for 1 h at 4 °C and the L-EV_Mreg_-containing pellet was resuspended in PBS and subjected to further analyses. Regarding Mreg and L-EV_Mreg_ recovery at day 7, typically around 16 × 10^6^ Mreg and 31 × 10^6^ L-EV_Mreg_ were obtained per 100-ml cultivation bag filled with 25ml culture medium.

### Automated cell and vesicle analysis

Basic parameters such the number of particles (cells or vesicles) were evaluated using a MOXI cell counter (Orflo, Ketchum, ID, USA) which analyzes membrane surrounded structures within a size between 3 and 25 µm based on the Coulter principle (Fig. 1B, histogram).

### In vitro T-cell activation assay

**Fig. 2:**
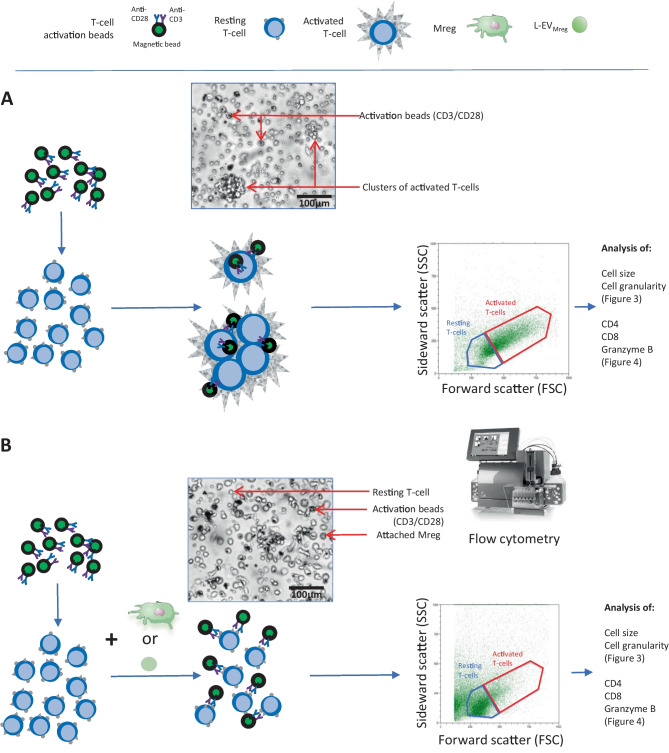
Schematic illustration of CD3/CD28-induced T-cell activation. **A** Administration of CD3/CD28 Dynabeads and subsequent binding to the corresponding surface receptors on resting T-cells lead to their activation. Using brightfield microscopy, activated T-cells dominate as clusters of large cells and are also detectable in the FSC/SSC flow cytometry blots. **B** Addition of Mreg or increasing concentrations of L-EV_Mreg_ to CD3/CD28-induced T-cells leads to inhibition of T-cell activation and lack of T-cell clustering

Employing the CD14^+^ monocyte isolation methodology detailed in this context, the selection column allowed the passage of exclusively CD14^−^ cells. These cells were harvested and employed as a source for T-cell activation assays (Fig. 1A). For the activation process, CD14^−^ cells were pre-incubated with CD3/CD28 activation beads (Dynabeads Human T activation, Gibco, MA, USA) for 15 min, following the manufacturer’s guidelines. Subsequently, CD14^−^ cells (0.35 × 10^6^/ml) were cultured either alone (activation control), in co-culture with Mreg (0.5 × 10^6^/ml), or with varying concentrations of L-EV_Mreg_ (0.8, 1.6, and 3.2 × 10^6^/ml). The decision for the used concentrations of L-EV_Mreg_ was based on the consideration that (i) the application of 0.5 × 10^6^ Mreg/ml resulted in a statistically significant reduction of T-cell activation, and (ii) the L-EV_Mreg_/Mreg ratio at the end of the differentiation period was 1.97 ± 0.64. After a co-incubation period of 3–5 days, supernatants containing non-adherent cells (including T-cells) were collected. The suspension was cleared of magnetic beads by positioning it near a magnetic field (MiniMACS™ separator, Miltenyi Biotec, Germany) for 5 min. Subsequently, the T-cells underwent flow cytometric analysis as described below (Fig. 2). For each T-cell activation assay, Mreg and L-EV_Mreg_ from the same preparation/batch were used. Mreg or L-EV_Mreg_ were not pooled.

### Flow cytometry

Cell or vesicle surface markers were analyzed in each L-EV_Mreg_/Mreg preparation by flow cytometry before cells and vesicles were used for further experiments. Standard markers that were examined are CD31, CD206, CD11c, CD86, CD14, CD16, CD103, CD38, CD45, LAMP-1, CD9, CD63, and CD81. For details, refer to our previous publication [8].

Flow cytometry was performed using the MACS Q10™ cytometer (Miltenyi Biotec, Germany). Fluorescein isothiocyanate (FITC)–conjugated specific antibodies including anti-CD4 and anti-mouse IgG1κ (both from BD Biosciences), phycoerythrin (PE)-conjugated anti-granzyme B, anti-mouse IgG1κ (both from Invitrogen), and allophycocyanin (APC)-conjugated anti-CD8 and anti-mouse IgG1κ (both from BD Biosciences) were used. To assess viability, 7-AAD (BD Biosciences) staining was used.

Cells were incubated with anti-CD4 and anti-CD8 antibodies or their respective isotype controls for 30 min at 4 °C. For analyses of intracellular granzyme B, cells were fixed, permeabilized with eBioscience Fixation/Permeabilization Concentrate (Invitrogen, USA), and incubated with granzyme B or the isotype control antibodies for 20 min at 4 °C. The gating strategy consisted of selecting the respective singlet population on a FSC-A/FSC-H plot, choosing the T-cell population based on size and granularity (FSC/SSC profile), and evaluating the respective target protein taking into account the isotype control signal of 7-AAD-negative (living) cells.

To evaluate the exposure of PS on the outer cell membrane by flow cytometry, an annexin V-FITC kit (Miltenyi Biotec, Germany) was employed following the manufacturer’s instructions. Briefly, freshly isolated cells were incubated for 20 min with annexin V buffer and washed twice in the same buffer, and the final pellet was separated into four groups to incubate the cells (i) without any fluorochrome, (ii) with annexin V, (iii) with propidium iodide (PI), or (iv) with annexin V and PI. Annexin V was added and incubated for 20 min, while PI was added shortly before performing flow cytometry scans. Co-staining of cells with PI and annexin V was considered indicative of dead cells.

### Fluorescence microscopy

For the visualization of PS in the outer cell/vesicle membrane, L-EV_Mreg_-containing Mreg pellets were resuspended in a buffer consisting of 10 mM HEPES and 145 mM NaCl. The resuspended Mreg and L-EV_Mreg_ were supplemented with 10 µM of PSVue480 (Molecular Targeting Technologies, West Chester, PA, USA) and 0.2 ng/ml of Hoechst33342 for nuclei staining. The mixture was then kept in the dark at 37 °C for 15 min before being centrifuged at 500xg for 5 min. The resulting pellets were then resuspended in HEPES buffer. A small quantity of the resuspended Mreg and L-EV_Mreg_ was placed on a glass slide, covered with a glass coverslip, and immediately examined. The analysis was carried out using a Leica DM2000 LED fluorescent microscope equipped with DAPI, L5, and rhodamine filter cubes; a HC PL FLUOTAR × 40/0.80 objective; and a Leica DFC7000 T fluorescence camera, using the Image Overlay software.

### Statistics

All experiments were carried out with cells and L-EV_Mreg_ derived from at least 5 healthy donors. The statistics software GraphPad Prism 5.01 for windows (GraphPad Software, San Diego, USA) was used to compare groups. All data were tested for normality using the Kolmogorov-Smirnov test. In cases normality was not obtained, the data were transformed (arcsin of square root of *x*) and analyzed using one-way ANOVA with Tukey test. A *P*-value < 0.05 was considered significant. All values are expressed as mean ± standard error mean (SEM).

## Results

### Basic characteristics of Mreg and L-EV_Mreg_

The present study involves the characterization of monocyte-derived Mreg and L-EV_Mreg_ obtained after 7 days of differentiation. Mreg recovery rate at harvest and viability were 71.83 ± 5.91% and 83.54 ± 4.12%, respectively (data not shown). Visually, Mreg did not exhibit any signs of apoptosis such as blebbing or shrinkage and showed a typical morphology upon attachment to the culture well (Fig. 1A). Flow cytometry data revealed Mreg as highly viable granulated cell population, whereas L-EV_Mreg_ dominated as a defined, less granulated population lacking nuclei (Figs. 1B and 4). Using the Coulter principle–based MOXI cell counter, the presence of two distinct populations, one of 7.47 ± 0.75µm corresponding to L-EV_Mreg_ and the other of 13.73 ± 1.33µm corresponding to Mreg, could be confirmed (Fig. 1B). L-EV_Mreg_ are only present in Mreg cultures; the culture medium as well as the added human AB serum is devoid of L-EV (data not shown). A characterization of Mreg and L-EV_Mreg_ following the MISEV criteria [9] has been published by our group recently [8].

### Effects of Mreg and L-EV_Mreg_ on CD3/CD28-induced T-cell activation

T-cells can be activated in vitro through the simultaneous binding of CD3 and CD28 to the corresponding receptors on the T-cell surface which is achieved by the addition of CD3/CD28 coated beads to the respective lymphocyte preparations [13]. Activated T-cells are easily identified in culture by an increase in cell size and granularity and the presence of cell accumulations. While clusters of activated T-cells are already visible using conventional brightfield microscopy, the increase in cell size and granularity can be evaluated by flow cytometry as a shift of the cell population toward higher FSC and SSC values. Using the described experimental setup (Fig. 2), we have analyzed the influence of Mreg and L-EV_Mreg_ on T-cell activation.

**Fig. 3:**
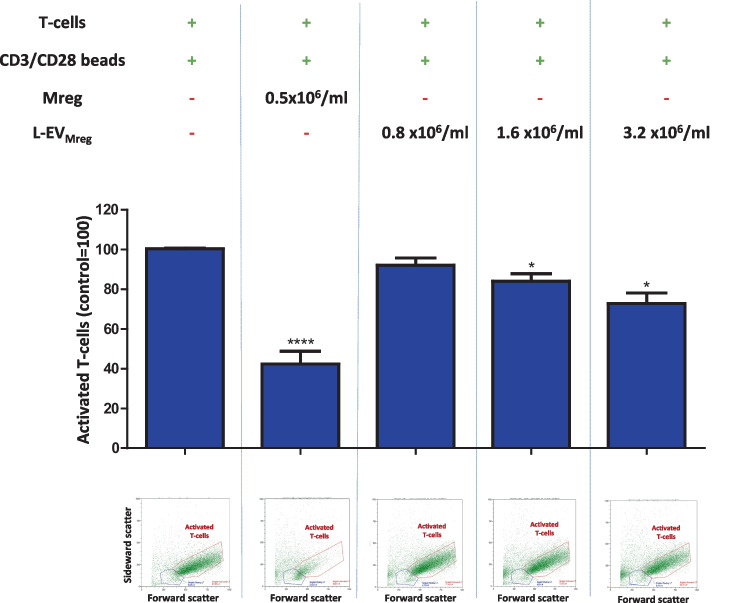
Effects of Mreg and L-EV_Mreg_ on the T-cell activation (cell size and cell granularity). Relative number of activated T-cells. A representative flow cytometry dot plot is shown below the respective columns. *P < 0.05; ****P < 0.0001 vs. activated T-cells (group: T-cells + CD3/CD28 beads)

In a first step, T-cell activation was estimated based on an increase in cell size (FSC in flow cytometry) and cell granularity (SSC in flow cytometry). Addition of Mreg resulted in a significant reduction in the number of activated T-cells compared to the control (CD3/CD28 activated T-cell control, 100%; Mreg, 42.42 ± 5.99%; *P* < 0.0001; Fig. 3). The addition of L-EV_Mreg_ resulted in a dose-dependent inhibition of T-cell activation (CD3/CD28 activated T-cell control, 100%; 0.8 × 10^6^ L-EV_Mreg_/ml, 92.17 ± 3.29%, *P* > 0.05; 1.6 × 10^6^ L-EV_Mreg_/ml, 84.00 ± 3.53%, *P* < 0.05; 3.2 × 10^6^ L-EV_Mreg_/ml, 72.81 ± 4.80%, *P* < 0.05; Fig. 3).

Since activated T-cells express large amounts of intracellular granzyme B [14, 15], this enzyme was used as additional reliable intracellular marker to quantify the number of activated T-cells.

**Fig. 4:**
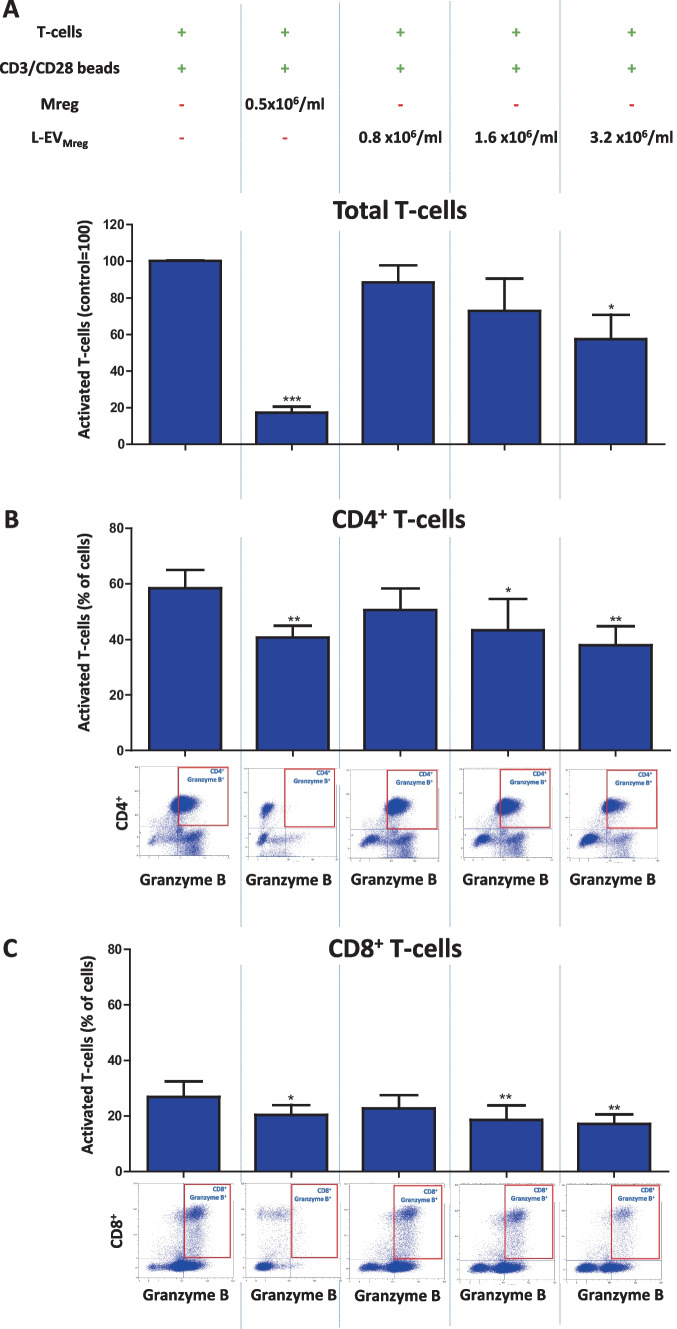
Effects of Mreg and L-EV_Mreg_ on the T-cell activation (intracellular granzyme B). **A** Relative number of activated T-cells. **B** Activated ${\mathrm{CD}4}^{+}$ T-cells. **C** Activated ${\mathrm{CD}8}^{+}$ T-cells. A representative flow cytometry dot plot is shown below the respective columns in **B** and **C**. *P < 0.05; **P < 0.01; ***P < 0.001 vs. activated T-cells (group: T-cells + CD3/CD28 beads)

Our results show that the addition of Mreg causes a significant reduction in the number of activated T-cells compared to the control (CD3/CD28 activated T-cell control, 100%; Mreg, 17.29 ± 3.35%; *P* < 0.001; Fig. 4A). The addition of L-EV_Mreg_ resulted in a dose-dependent inhibition of T-cell activation, although a statistically significant effect could only be achieved with the highest concentration of L-EV_Mreg_ (CD3/CD28 activated T-cell control, 100%; 0.8 × 10^6^ L-EV_Mreg_/ml, 88.41 ± 9.38%, *P* > 0.05; 1.6 × 10^6^ L-EV_Mreg_/ml, 72.95 ± 17.51%, *P* > 0.05; 3.2 × 10^6^ L-EV_Mreg_/ml, 57.48 ± 13.30%, *P* < 0.05; Fig. 4A).

Additional stratification of T-cells into CD4^+^ helper T-cells and CD8^+^ cytotoxic T-cells showed that both Mreg and L-EV_Mreg_ were able to inhibit CD3/CD28-induced activation of both T-cell subpopulations: CD4^+^ T-cells: CD3/CD28 activated CD4^+^ T-cell control, 58.42 ± 6.58% (granzyme B–positive cells); Mreg, 40.68 ± 4.19%, *P* < 0.01; 0.8 × 10^6^ L-EV_Mreg_/ml, 50.61 ± 7.72%, *P* > 0.05; 1.6 × 10^6^ L-EV_Mreg_/ml, 43.33 ± 11.18%, *P* < 0.05; 3.2 × 10^6^ L-EV_Mreg_/ml, 37.93 ± 6.78%, *P* < 0.01; Fig. 4B. CD8^+^ T-cells: CD3/CD28 activated CD8^+^ T-cell control, 26.89 ± 5.51% (granzyme B–positive cells); Mreg, 20.43 ± 3.48%, *P* < 0.05; 0.8 × 10^6^ L-EV_Mreg_/ml, 22.71 ± 4.77%, *P* > 0.05; 1.6 × 10^6^ L-EV_Mreg_/ml, 18.61 ± 5.15%, *P* < 0.01; 3.2 × 10^6^ L-EV_Mreg_/ml, 17.16 ± 3.37%, *P* < 0.01; Fig. 4C.

Another marker for the assessment of T-cell activation is CD25 which is upregulated on the surface of activated T-cells and serves as a high-affinity receptor for interleukin-2 (IL-2), a key cytokine involved in T-cell proliferation and survival [16]. In addition to the granzyme B evaluation, quantification of the number of CD25-positive T-cells also confirmed the above described effects of T-cell inhibition by Mreg and L-EV_Mreg_ (data not shown).

### Phosphatidylserine exposure on the membrane of Mreg and L-EV_Mreg_

**Fig. 5:**
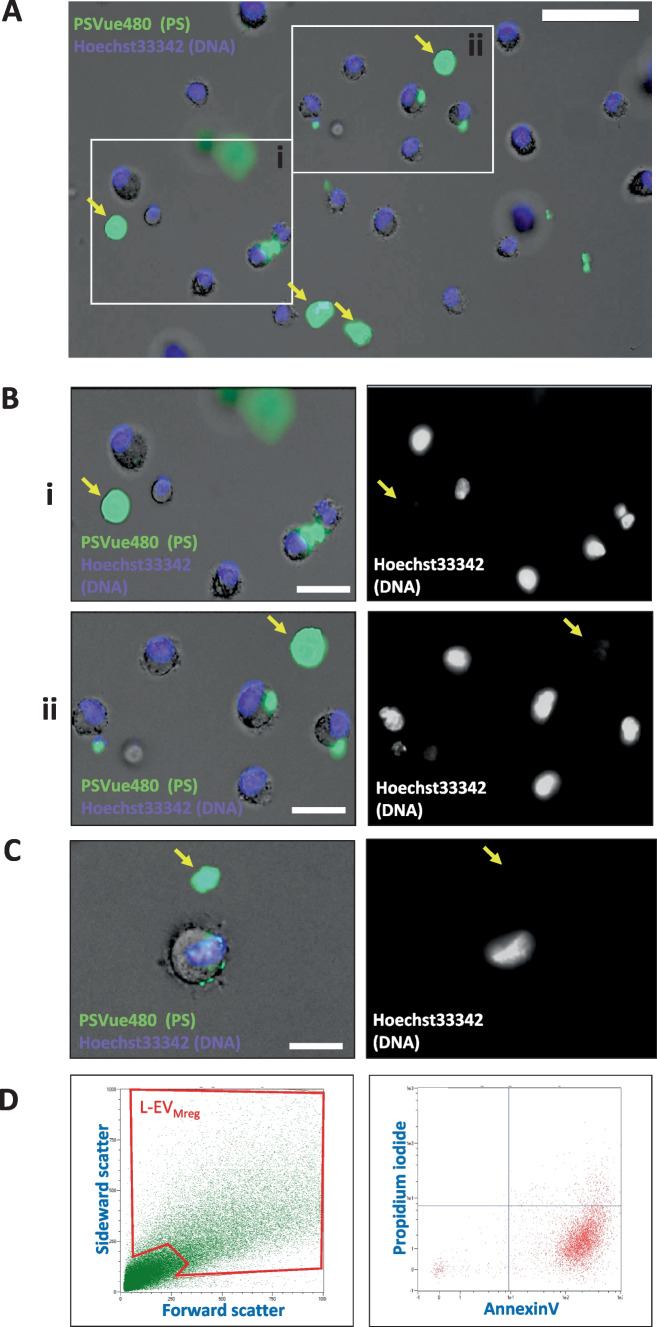
Phosphatidylserine exposure on the membrane of Mreg and L-EV_Mreg_. **A** Cells and vesicles were stained with Hoechst33342 (violet) and PSVue480 (green) to label DNA and PS, respectively. Signals were merged on the brightfield background to distinguish morphological features. Yellow arrows show L-EV_Mreg_. Bar denotes 50 µm. **B** Left: magnified views of two selected areas from **A**. Right: same area as on the left showing only the nuclei staining. Yellow arrows denote L-EV_Mreg_. Note that L-EV_Mreg_ lack nuclei and are positive for PS, while Mreg contain nuclei and are either negative for PS or reveal PS to be restricted to only a confined region of the cell surface. Bar denotes 20 µm. **C** Typical PS staining of a Mreg cell and one L-EV_Mreg_. Yellow arrows denote L-EV_Mreg_. Bar denotes 15 µm. **D** Left: Flow cytometry and gating (red box) of L-EV_Mreg_. Right: Gated L-EV_Mreg_ are positive for PS (annexin V staining) and negative for nucleic acids (propidium iodide staining)

As the exposure of phosphatidylserine (PS) by non-apoptotic cells has been recently recognized as an immunomodulatory mechanism [17] and PS is present in the cell membrane of non-apoptotic macrophages [18], we decided to analyze whether PS is also detectable in the outer lipid bilayer of Mreg and L-EV_Mreg_. Nuclear staining for viable cells and PS staining were performed on Mreg and L-EV_Mreg_. The results show that Mreg are either negative for PS or that PS is restricted to a confined region of the cell surface. In contrast to Mreg, L-EV_Mreg_ lack nuclei and show strong signals for PS on the entire surface (Fig. 5A–C). Extended analysis of the vesicular fraction by cytometry confirmed the fluorescence microscopy findings defining L-EV_Mreg_ as PS-positive vesicular structures devoid of nuclei.

## Discussion

Eukaryotic cells release extracellular vesicles (EV) into their microenvironment, which can vary in size and cargo composition [1, 2, 19]. EV transport a plethora of diverse biological molecules including lipids, carbohydrates, proteins, and RNAs, and their characteristics may vary depending on the conditions they are exposed to, allowing them to effectively transmit functional content and signals between cells [1, 2, 5, 20].

Macrophages are a type of white blood cell that play diverse roles in the immune response, including phagocytosis, antigen presentation, and regulation of inflammation. They can be polarized into different subsets, such as M1 and M2, which exhibit distinct functional and phenotypic characteristics [21, 22]. Macrophage-derived EV [23] have been found to play a role in numerous physiological and pathological pathways [24, 25] and to modulate immune responses [25–27].

Regulatory macrophages (Mreg), which in particular possess characteristics of anti-inflammatory M2 macrophages, can be generated from peripheral blood monocytes under defined growth factor-induced culture conditions [28–30]. Mreg secrete pro-angiogenic factors as well as various cytokines and have already been used in cell therapy–based trials to reduce organ rejection after kidney transplantation [12, 30]. In this context, intravenously applied Mreg minimized the burden of general immunosuppression after organ transplantation [12] and the underlying mechanisms may involve a Mreg-mediated inhibition of immune cell activation [10].

In our recent work, we have shown that monocyte-derived Mreg also secrete large EV (L-EV_Mreg_), with an average diameter of 7.5 µm and an average volume of 0.22 pl, which contain pro-angiogenic molecules and induce angiogenesis and wound healing in vitro [8]. Here, we demonstrate for the first time that L-EV_Mreg_ are also able to attenuate CD3/CD28-induced activation of CD4^+^ and CD8^+^ T-cells.

T-cells play a crucial role in the immune system function by recognizing and responding to foreign antigens. Upon activation, they proliferate and differentiate into effector T-cells that coordinate an immune response against the pathogen [31]. Effector T-cells can be divided into helper and cytotoxic T-cells [32, 33]. Helper T-cells also known as CD4^+^ T-cells recognize and bind to antigen-presenting cells and provide help to other immune cells, such as B-cells and macrophages, to mount an effective immune response. They secrete cytokines that activate and coordinate the immune response against the pathogen [34, 35]. In contrast, cytotoxic T-cells, also known as CD8^+^ T-cells, recognize and kill infected or cancerous cells through the release of cytotoxic molecules. They play a crucial role in the elimination of intracellular pathogens and tumor cells [36]. Dysregulation of T-cell function can lead to various immune-related disorders, including autoimmune diseases and cancer [37–39].

Utilizing cytometric analyses, the current investigation revealed that both Mreg and L-EV_Mreg_ possess the ability to attenuate T-cell activation induced by CD3/CD28 stimulation. The suppressive potential of Mreg was found to be considerably greater compared to L-EV_Mreg_; however, the latter still exhibited a statistically significant level of T-cell inhibition, particularly at the highest concentration of 3.2 × 10^6^ L-EV_Mreg_/ml employed. Despite the inferiority of L-EV_Mreg_ in terms of T-cell inhibition when compared to Mreg, L-EV_Mreg_ present numerous advantages for potential clinical applications: one major advantage of L-EV_Mreg_ is their lack of a cellular nucleus and inability to replicate. Consequently, L-EV_Mreg_ do not possess characteristics of living organisms, which reduces the likelihood of side effects that are commonly associated with the administration of autologous or allogeneic cells in a clinical context. Moreover, large quantities of L-EV_Mreg_ can be obtained from Mreg cultures as they are continually produced by Mreg and storage and handling of L-EV_Mreg_ are also considerably more convenient than those of vital Mreg cells. Finally, since L-EV_Mreg_ do not fall under the classification of Advanced Therapy Medicinal Products (ATMPs), they are exempt from the corresponding regulatory restrictions. This enables a faster and more convenient transition of a possible L-EV_Mreg_ treatment into clinical applications.

Regarding the eukaryotic lipid bilayer cell membrane, it is widely accepted that apoptosis triggers the exposure of PS on the outer membrane surface [40]. However, this distribution pattern of PS can also be observed in non-apoptotic cells [41] and it was shown that PS expressed on the surface of human exosomes is linked to T-cell immunosuppression [40, 42]. Therefore, it could be proposed that the exposure of PS in the exoplasmic leaflet of Mreg and L-EV_Mreg_ described in the present work may be involved in the Mreg- and L-EV_Mreg_-mediated attenuation of T-cell activation. Also, the PD-1/PD-L1 system, which plays an important role in regulating several components of the immune system [43–45], might be involved in T-cell regulation. Preliminary findings of our group showed that 82.6 ± 3.8% of Mreg and 22.0 ± 6.1% of L-EV_Mreg_ are positive for PD-L1, the ligand for PD-1 which is expressed on the surface of T-cells [43, 46]. Although the above-mentioned potential mechanisms of T-cell inhibition by L-EV_Mreg_ are scientifically of interest, they are of only secondary importance for the potential clinical application of L-EV_Mreg_ and were not the main objective of our present work.

It should be noted that despite intensive characterization of L-EV_Mreg_, their categorization into the different classes of EV is not fully established at this stage. However, there are several similarities between exophers and L-EV_Mreg_ [47]. Exophers are several micrometers in size and have been demonstrated to detach from cells within a matter of hours [48]. Unfortunately, we are unable to investigate this characteristic trait of exophers in the context of L-EV_Mreg_. Throughout the differentiation phase, Mreg remain within the culture bags inside the incubator, rendering them inaccessible to morphological analyses. Furthermore, even the simple act of transferring the bags from the incubator to the microscope is likely to dissolve any potential connection between the exophers and their parent cells. However, exophers typically possess a lipid bilayer and exhibit phosphatidylserine on their surface, thereby sharing certain characteristics with the L-EV_Mreg_ described in our study [48].

In conclusion, the present study has revealed the novel finding that PS positive L-EV_Mreg_ can suppress both CD4^+^ and CD8^+^ T-cells, suggesting the potential clinical use of L-EV_Mreg_ in various diseases associated with increased T-cell activity, including multiple sclerosis, rheumatoid arthritis, type 1 diabetes, allergic reactions, and transplant rejection.

## Data Availability

The data that support the findings of this study are available from the corresponding author upon reasonable request.
